# Supplementary material for: Separation of α-Lactalbumin Enriched Fraction from Bovine Native Whey Concentrate by Combining Membrane and High-Pressure Processing
Source: Foods. 2023 Jan 19;12(3):480. doi: 10.3390/foods12030480 (PMC9914712; doi:10.3390/foods12030480)
Supplement: Supplementary file 1 [file foods-12-00480-s001.zip › foods-2143772-supplementary.pdf]

foods

Supplementary Materials

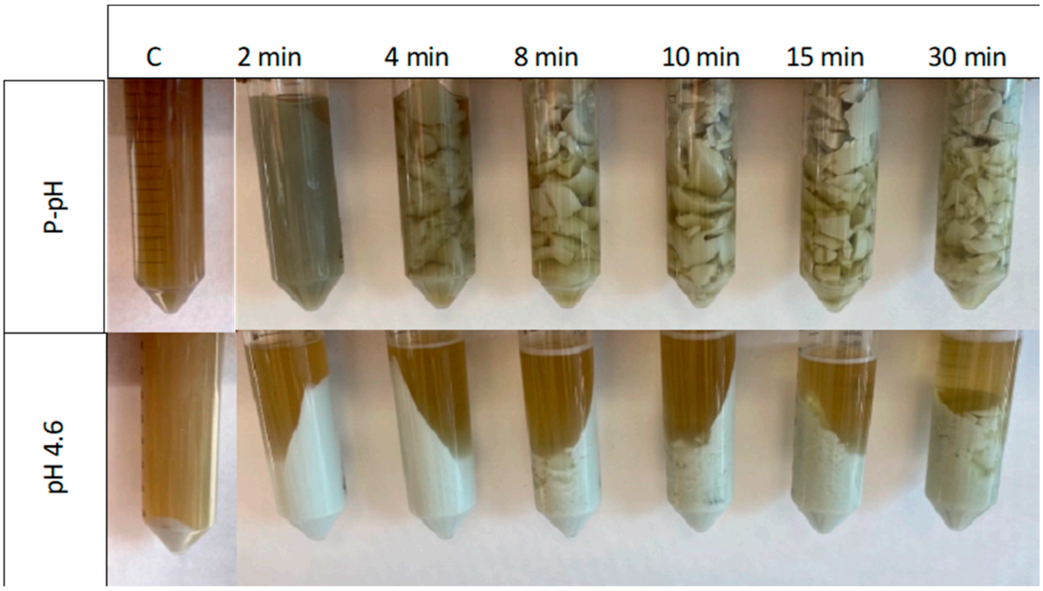

Figure S1. Visual appearance of native concentrated whey after high pressure processing (HPP) (600 MPa, 23ºC) at physiological (P-pH) and 4.6 pH for 0 (control) 2, 4, 8, 10, 15 and 30 min of treatment time.

Table S1. Predictive regression models coefficients obtained from the first set of experiments. ($P^* = P - 397.11$; $t^* = t - 240$, and $T^* = T - 22.79$). ($P^+ = P - 377.25$; $t^+ = t - 228$, and $T^+ = T - 21.65$) and lack of fit of the models. RSME: Root Mean Square Error. P = HPP pressure level; T = initial sample temperature; t = HPP processing time.

pH	Parameter	Predictive equation	R^2	RSME	Lack of fit Prob > F
Physiological	a-La yield	$163.75 - 0.09 \cdot P - 0.06 \cdot t - 0.63 \cdot T + (-0.00040 \cdot P^* \cdot P^*) + (-0.0004 \cdot t^* \cdot P^*) + (-0.00003 \cdot t^* \cdot t^*) + (-0.0056 \cdot T^* \cdot P^*) + (0.00036 \cdot T^* \cdot t^*) + (-0.0278 \cdot T^* \cdot T^*)$	0.92	9.33	0.4919
	a-La purifications degree	$-18.64 + 0.07 \cdot P - 0.04 \cdot t - 0.61 \cdot T + (0.00014 \cdot P^+ \cdot P^+) + (0.00012 \cdot t^+ \cdot P^+) + (-2.872 \cdot t^+ \cdot t^+) + (0.0023 \cdot T^+ \cdot P^+) + (0.0002 \cdot t^+ \cdot T^+) + (-0.0026 \cdot T^+ \cdot T^+)$	0.92	5.91	<0.0001
Acidified	a-La yield	$85.65 - 0.08 \cdot P - 0.007 \cdot t - 0.26 \cdot T + (-0.00004 \cdot P^* \cdot P^*) + (-0.00006 \cdot t^* \cdot P^*) + (0.00024 \cdot t^* \cdot t^*) + (0.0003 \cdot T^* \cdot P^*) + (-0.00078 \cdot T^* \cdot t^*) + (-0.0098 \cdot T^* \cdot T^*)$	0.86	7.22	0.2499
	a-La purifications degree	$-27.99 + 0.13 \cdot P + 0.03 \cdot t + 0.34 \cdot T + (0.00036 \cdot P^* \cdot P^*) + (0.000052 \cdot t^* \cdot P^*) + (-0.00008 \cdot t^* \cdot t^*) + (0.0012 \cdot T^* \cdot P^*) + (-0.0011 \cdot t^* \cdot T^*) + (-0.004 \cdot T^* \cdot T^*)$	0.99	3.54	0.0002

Table S2. Second set of experiments. Protein concentrations and main process performance parameters of the a-Lactalbumin (a-La) enriched fractions (supernatants) after different HPP treatments. Each value is expressed as mean value ± SD (*n* = 4).

pH	Time (min)	Concentration of a-La (mg/mL)	Concentration of b-Lg A (mg/mL)	Concentration of b-Lg B (mg/mL)	a-La yield (%)	a-La yield (%)$^+$	a-La purification degree (%)	b-Lg A precipitation degree (%)	b-Lg B precipitation degree (%)
6.8	Control	28.75 ± 2.20^{ab}	30.43 ± 8.64^{a}	75.79 ± 5.37^{a}	-	-	21.93 ± 0.70^{i}	-	-
	2	28.57 ± 5.87^{a}	12.46 ± 3.62^{b}	35.81 ± 6.94^{b}	$55.55 \pm 12.58^{b*}$	103.22 ± 5.65^{a}	36.67 ± 2.05^{h}	$79.67 \pm 12.10^{c*}$	$80.99 \pm 11.71^{c*}$
	4	24.05 ± 5.95^{abc}	8.29 ± 3.36^{b}	25.82 ± 6.18^{c}	$49.13 \pm 10.46^{bc*}$	97.67 ± 5.29^{abc}	43.97 ± 2.78^{g}	$87.80 \pm 7.08^{bc*}$	$87.88 \pm 7.00^{bc*}$
	8	20.74 ± 4.51^{abc}	3.85 ± 0.92^{c}	15.67 ± 5.28^{d}	$31.53 \pm 4.31^{def*}$	$82.31 \pm 8.59^{bc*}$	58.54 ± 4.28^{f}	$94.27 \pm 3.40^{ab*}$	$95.80 \pm 2.32^{ab*}$
	10	20.28 ± 5.30^{bc}	3.24 ± 1.95^{c}	13.41 ± 5.11^{de}	$27.37 \pm 3.89^{efg*}$	$79.27 \pm 13.95^{c*}$	60.39 ± 6.70^{f}	$95.51 \pm 2.72^{ab*}$	$96.83 \pm 2.42^{a*}$
	15	17.16 ± 6.38^{cd}	1.75 ± 1.12^{c}	6.09 ± 4.04^{ef}	$19.51 \pm 5.56^{fg*}$	$50.15 \pm 10.87^{d*}$	74.54 ± 9.34^{e}	$98.75 \pm 1.06^{a*}$	$98.87 \pm 0.92^{a*}$
	30	11.40 ± 6.44^{d}	1.64 ± 0.55^{c}	3.23 ± 2.08^{f}	$13.48 \pm 6.18^{g*}$	$29.29 \pm 12.12^{e*}$	79.32 ± 5.68^{cde}	$99.44 \pm 0.36^{a*}$	$99.29 \pm 0.65^{a*}$
4.6	0	23.88 ± 3.65^{abc}	24.33 ± 5.04^{a}	74.60 ± 15.75^{a}	$85.83 \pm 7.41^{a*}$	$103.88 \pm 5.57^{a*}$	21.68 ± 0.55^{i}	$24.49 \pm 12.25^{d*}$	$18.86 \pm 10.02^{d*}$
	2	23.21 ± 3.18^{abc}	2.26 ± 0.50^{c}	5.04 ± 1.48^{ef}	$42.73 \pm 6.19^{bcde*}$	98.38 ± 3.50^{ab}	78.10 ± 2.48^{de}	$97.37 \pm 0.88^{ab*}$	$96.39 \pm 1.42^{a*}$
	4	23.52 ± 3.05^{abc}	2.48 ± 1.58^{c}	6.33 ± 5.16^{ef}	$46.16 \pm 6.98^{bcd*}$	99.24 ± 2.11^{ab}	80.21 ± 2.04^{cde}	$95.91 \pm 4.38^{ab*}$	$95.34 \pm 4.35^{ab*}$
	8	23.04 ± 3.52^{abc}	1.41 ± 0.26^{c}	3.15 ± 0.52^{f}	$46.45 \pm 11.03^{bcd*}$	99.04 ± 5.57^{ab}	84.82 ± 2.03^{bcd}	$98.11 \pm 0.83^{a*}$	$97.47 \pm 1.24^{a*}$
	10	22.09 ± 3.13^{abc}	1.33 ± 0.23^{c}	2.81 ± 0.61^{f}	$45.95 \pm 7.04^{bcd*}$	96.85 ± 5.63^{abc}	85.90 ± 1.98^{abc}	$98.26 \pm 0.73^{a*}$	$97.55 \pm 1.06^{a*}$
	15	21.26 ± 2.62^{abc}	1.05 ± 0.16^{c}	2.16 ± 0.49^{f}	$41.94 \pm 7.94^{bcde*}$	$91.87 \pm 4.21^{abc*}$	88.16 ± 1.61^{ab}	$98.71 \pm 0.55^{a*}$	$98.13 \pm 0.85^{a*}$
	30	19.14 ± 2.97^{cd}	0.72 ± 0.17^{c}	1.17 ± 0.41^{f}	$35.88 \pm 8.25^{cde*}$	$82.29 \pm 1.33^{bc*}$	92.01 ± 1.71^{a}	$99.34 \pm 0.31^{a*}$	$98.81 \pm 0.52^{a*}$

$^{a-i}$Mean values in the same column with different superscripts differ (P < 0.05) according to Tukey test. *Indicate significant differences of the mean if compared to the control (T-test). $^+$Following Marciniak et al. [36]
